# Machine Learning-Based Evaluation on Craniodentofacial Morphological Harmony of Patients After Orthodontic Treatment

**DOI:** 10.3389/fphys.2022.862847

**Published:** 2022-05-09

**Authors:** Xin Wang, Xiaoke Zhao, Guangying Song, Jianwei Niu, Tianmin Xu

**Affiliations:** ^1^ Department of Orthodontics, Peking University School and Hospital of Stomatology, Beijing, China; ^2^ State Key Laboratory of Virtual Reality Technology and Systems, School of Computer Science and Engineering, Beihang University, Beijing, China; ^3^ Beijing Advanced Innovation Center for Big Data and Brain Computing (BDBC), Beihang University, Beijing, China; ^4^ Hangzhou Innovation Research Institute, Beihang University, Beijing, China; ^5^ NHC Research Center of Engineering and Technology for Computerized Dentistry, Beijing, China

**Keywords:** cephalometric analysis, facial harmony, machine learning, malocclusion, orthodontic treatment

## Abstract

**Objectives:** Machine learning is increasingly being used in the medical field. Based on machine learning models, the present study aims to improve the prediction performance of craniodentofacial morphological harmony judgment after orthodontic treatment and to determine the most significant factors.

**Methods:** A dataset of 180 subjects was randomly selected from a large sample of 3,706 finished orthodontic cases from six top orthodontic treatment centers around China. Thirteen algorithms were used to predict the value of the cephalometric morphological harmony score of each subject and to search for the optimal model. Based on the feature importance ranking and by removing features, the regression models of machine learning (including the Adaboost, ExtraTree, XGBoost, and linear regression models) were used to predict and compare the score of harmony for each subject from the dataset with cross validations. By analyzing the prediction values, the most optimal model and the most significant cephalometric characteristics were determined.

**Results:** When nine features were included, the performance of the XGBoost regression model was MAE = 0.267, RMSE = 0.341, and Pearson correlation coefficient = 0.683, which indicated that the XGBoost regression model exhibited the best fitting and predicting performance for craniodentofacial morphological harmony judgment. Nine cephalometric features including L1/NB (inclination of the lower central incisors), ANB (sagittal position between the maxilla and mandible), LL-EP (distance from the point of the prominence of the lower lip to the aesthetic plane), SN/OP (inclination of the occlusal plane), SNB (sagittal position of the mandible in relation to the cranial base), U1/SN (inclination of the upper incisors to the cranial base), L1-NB (protrusion of the lower central incisors), Ns-Prn-Pos (nasal protrusion), and U1/L1 (relationship between the protrusions of the upper and lower central incisors) were revealed to significantly influence the judgment.

**Conclusion:** The application of the XGBoost regression model enhanced the predictive ability regarding the craniodentofacial morphological harmony evaluation by experts after orthodontic treatment. Teeth position, teeth alignment, jaw position, and soft tissue morphology would be the most significant factors influencing the judgment. The methodology also provided guidance for the application of machine learning models to resolve medical problems characterized by limited sample size.

## 1 Introduction

Malocclusion has been considered to be highly prevalent and can affect oral and facial aesthetics as well as psychosocial wellbeing in the long term (Borzabadi-Farahani, 2012; Vellappally et al., 2014). It was claimed that the facial features, especially oral aesthetics, had the potential to influence self-perceived appearance, especially during the phase of life with intense social and affective interaction (Burden, 2007). Patients seeking orthodontic treatment aim to improve their dental aesthetics and facial balance (Turley, 2015; Singh et al., 2021). As a standardized method, cephalometric analysis is routinely used to investigate the interrelationship among craniofacial bony and soft tissue landmarks and is employed as a treatment planning and evaluation tool by orthodontists, based on cephalometric radiographs before and after orthodontic treatment. It is usually based on a comparison of the values obtained from certain measurements in a group of individuals with the average values from their populations, which is set as the normal or average value. The distance and angle deviations among cephalometric landmarks for patients are compared with this value to determine whether any skeletal or dental aberration exists. However, it might be misleading to practitioners that the process of orthodontic treatment is to “correct the abnormal values for each patient.” Actually, facial morphology varies in the size, shape, and position of the dentoskeletal structures for each individual, and the combinations of these morphological components are extremely diverse as well. It is important to understand that the aim of orthodontic treatment is to move teeth to a physiologically stable position and to balance the relationship within morphological components (Xu, 2017).

There has been a vast array of methods of cephalometric analysis during the past decades (Steiner, 1953; Downs, 1956; Ricketts, 1961; Tweed, 1969). Each method has some merits but may not be applicable in all cases. For a beginner in the field of orthodontics, it might be difficult to choose a certain method of cephalometric analysis that performs the best combination of the landmarks in a specific case. As practical training and clinical experience accumulate, clinicians will then be gradually familiar with each method of cephalometric analysis, which is useful in the understanding of specific morphological types and deformities. Based on accumulation by analyzing tens of thousands of patients, orthodontic experts could make validating judgments about patients’ morphological harmony when reading end-of-treatment cephalometric films. A panel of orthodontic experts from similar education and practicing backgrounds could reach an agreement on the perception of the harmonious relationship between the dentition and the facial configuration (Song et al., 2013, 2014).

When experts evaluate cephalometric morphological harmony, various landmarks and components of cephalometric films are concerned. However, among them, which are the most noteworthy and important features is unclear. The aim of this study is to determine the key characteristics of greatest concern when experts comprehensively value the various landmarks and components of cephalometric films. It may give researchers further ideas about how to improve the method that represents the evaluation and delivers the thoughts of experts more effectively and precisely. Solving these problems will also help beginners to obtain a thorough understanding of balanced dental, jaw, and facial relationships after orthodontic treatment and could further improve the existing evaluation system for orthodontic treatment outcomes.

Nowadays, machine learning is increasingly being used in the medical field ranging from medical image processing and the diagnosis of specific diseases to the broader tasks of decision support and outcome prediction (Hammond et al., 2001; Rubin et al., 2017; Torlay et al., 2017; Lee et al., 2018; Livne et al., 2018; Vaquerizo-Villar et al., 2018; Wang et al., 2018; Chang et al., 2019; Dinh et al., 2019; Xu et al., 2019; Suhail et al., 2020; Verma et al., 2020; You et al., 2020). However, machine learning methods are rarely applied to evaluate craniodentofacial morphological harmony after orthodontic treatment. The present study aims to predict the evaluation of orthodontic experts and focuses on predictive modeling of applications characterized by small datasets and real-numbered continuous outputs, based on machine learning models. Such tasks, in terms of predicting the evaluation of orthodontic experts, are mostly approached by using conventional multiple linear regression models, which are based on the assumptions of statistical independence of the input variables, linearity between dependent and independent variables, normality of the residuals, and the absence of endogenous variables. However, in many applications, particularly in those involving complex physiological parameters such as values of cephalometric analysis, these assumptions are often violated (Takada et al., 2000; Lee et al., 2014, 2018). This situation would necessitate more sophisticated regression models such as machine learning, in which the system can constantly update the models through new samples to improve the efficiency and accuracy of evaluation.

In order to explore the best-fitted modeling to predict the evaluation of orthodontic experts, several such systems including linear models, SVMs, decision trees, ANNs (artificial neural networks), and ensemble models are considered in the present study. We compared the abovementioned five categories of machine learning models involving 13 algorithms and searched for the best-fitting model for further assessing craniodentofacial morphological harmony. Based on machine learning models, our study aims to improve the prediction performance of craniodentofacial morphological harmony judgement after orthodontic treatment and to determine the most significant factors that influence the craniodentofacial morphological harmony judgement by orthodontists.

## 2 Materials and Methods

### 2.1 Quantification of the Subjective Evaluation From Orthodontic Experts

By random stratified sampling, the dataset of 180 subjects was selected from 3,706 Chinese malocclusion patients and was analyzed with two stratified samples. One stratified sample consisted of 108 subjects from the large sample of 2,383 finished orthodontic cases in six orthodontic treatment centers around China (including the Peking University School of Stomatology, the West China College of Stomatology at Sichuan University, the School of Stomatology at the Fourth Military Medical University, the Beijing Stomatology Hospital and School of Stomatology at the Capital Medical University, the Stomatology Hospital at Nanjing Medical University, and the Hospital of Stomatology at Wuhan University). The other comprised 48 subjects from another large sample of 1, 323 finished cases in the Peking University School of Stomatology and 24 overlapping subjects randomly selected from the former samples. The posttreatment lateral cephalometric X-ray images of the former samples were evaluated by a panel of 69 judges, and the latter samples were evaluated by another panel of 36 judges. Satisfactory cases were assigned a value of “1” point, acceptable cases were given “2” points, and unacceptable cases were given “3” points. For each case, the final score was the average point of all scores by the judges.

The panel of judges was recommended by the six participating treatment centers. The inclusion criteria for judges were that each had

1) an MS or Ph.D. degree in orthodontics or experience as a research supervisor of orthodontic postgraduates
2) no less than 10 years of clinical experience in orthodontics
3) the academic rank of associate professor or above

The experts who eventually participated in this study ranged in age from 40 to 60 years. Of the 69 experts on the panel, 38 were men and 31 were women; of the 36 experts on the panel, 19 were men and 17 were women.

The overlapping 24 samples were used to verify the consistency of the judges, which had a good result. Specifically, the Pearson correlation analysis showed that the two panels of judges were significantly and positively correlated (r = 0.905, *p* < 0.01), and no significant difference was found (for the paired *t* test, *p*＞0.05; for the intraclass correlation coefficient, ICC = 0.902). Details of the original data are shown in Supplementary Material S1.

The whole study was performed in accordance with the Declaration of Helsinki for research involving human subjects and reviewed and approved by the Ethics and Research Committee, Peking University School and Hospital of Stomatology (PKUSSIRB-201947092).

### 2.2 Cephalometric Analysis

**FIGURE 1:**
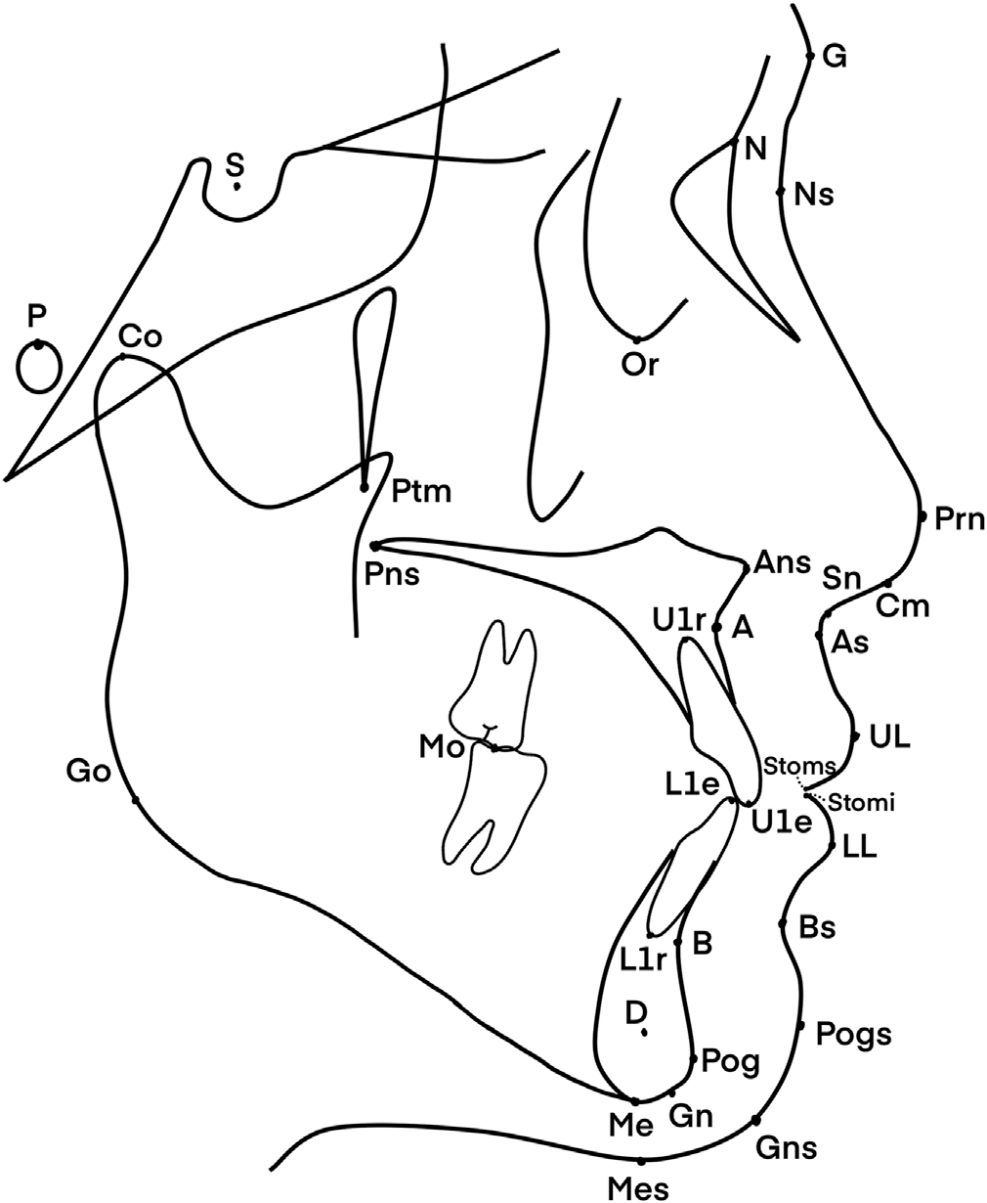
Landmarks of the lateral cephalogram.

**FIGURE 2:**
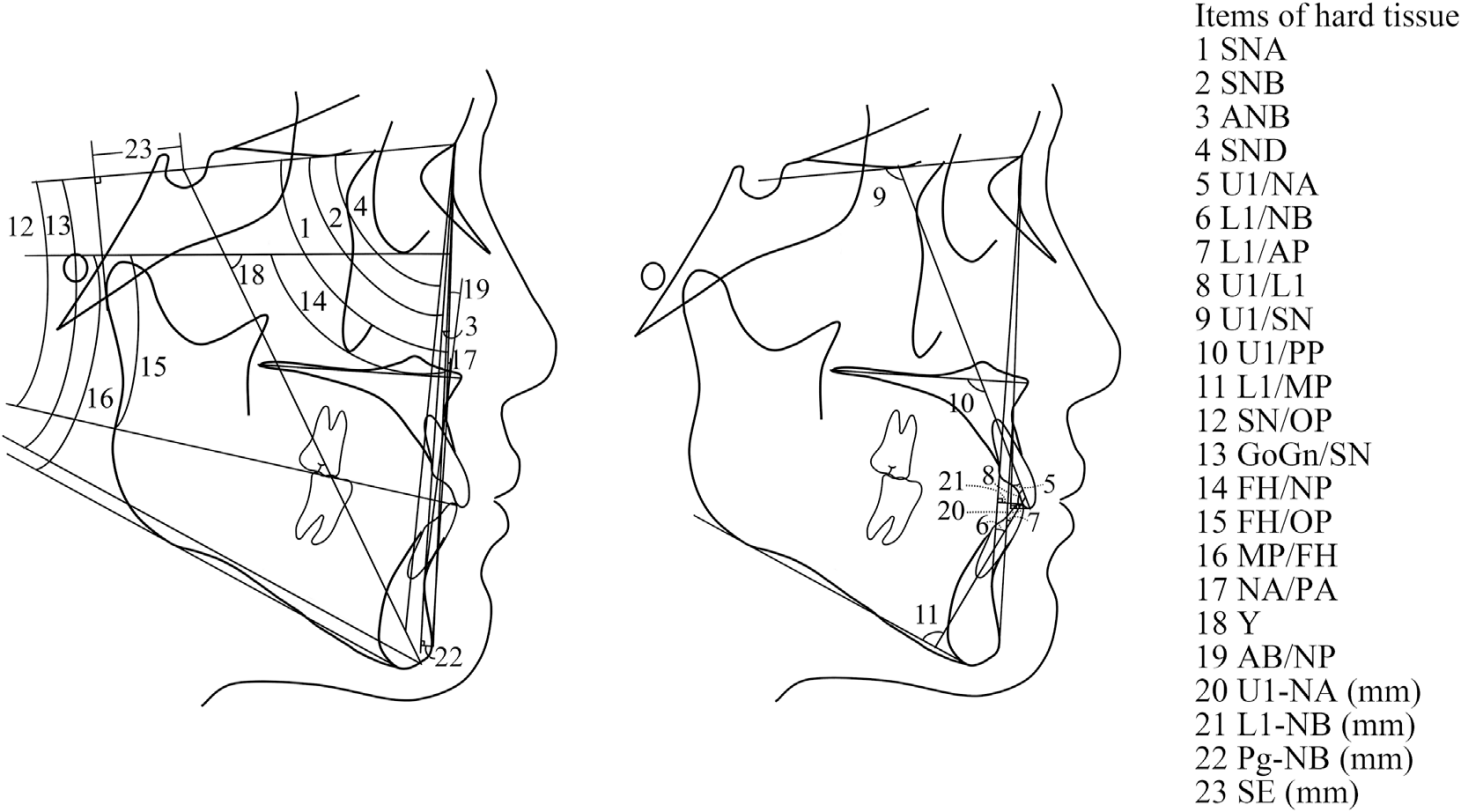
Cephalometric measurements of hard tissue.

**FIGURE 3:**
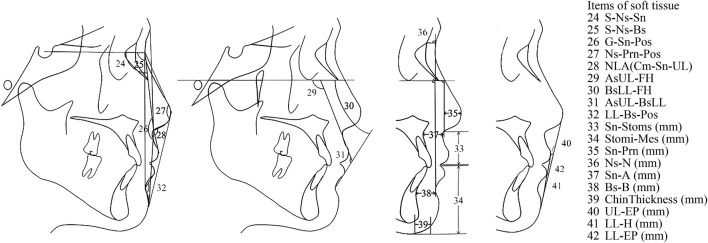
Cephalometric measurements of soft tissue.

The input data under consideration were derived from the anatomy of the patients, as shown in Figure 1. They were based on the 42 cephalometric features, which are shown and defined in Table 1. The cephalometric features were measured by three practitioners who were trained at the Peking University School of Stomatology. The lateral cephalogram landmarks and cephalometric measurement items were as in Figures 1–3 and Table 1.

**TABLE 1:** Definitions of the 42 cephalometric features.

No.	Cephalometric variables	Definition
1	SNA	Anteroposterior position of the maxilla to the anterior cranial base (degrees)
2	SNB	Anteroposterior position of the mandible to the anterior cranial base (degrees)
3	ANB	The angle between Down’s points A and B (degrees)
4	SND	The angle between the SN and ND line (degrees)
5	U1/NA	The angle between the line through the long axis of the upper central incisor and the NA line (degrees)
6	L1/NB	The angle between the line through the long axis of the lower central incisor and NB line (degrees)
7	L1/AP	The angle between the line through the long axis of the lower central incisor and the AP line (degrees)
8	U1/L1	The angle between the line through the long axis of the upper and lower central incisors (degrees)
9	U1/SN	The angle between the ling through the long axis of the upper central incisor and SN line (degrees)
10	U1/PP	The angle between the line through the long axis of the upper central incisor and palatal plane (degrees)
11	L1/MP	The angle between the line through the long axis of the lower central incisor and mandibular plane (degrees)
12	SN/OP	The angle between the SN line and occlusal plane (degrees)
13	GoGn/SN	The angle between the SN and GoGn line (degrees)
14	FH/NP	The angle between the Frankfort horizontal plane and NP line (degrees)
15	FH/OP	The angle between the Frankfort horizontal plane and occlusal Plane (degrees)
16	MP/FH	The angle between the mandibular plane and Frankfort horizontal plane (degrees)
17	NA/PA	The angle between the NA and PA line (degrees)
18	Y	Sella gnathion to the Frankfort horizontal plane (degrees)
19	AB/NP	The angle between the AB and NP line (degrees)
20	U1-NA	The perpendicular distance from U1 (incision superius) to the NA line (mm)
21	L1-NB	The perpendicular distance from L1 (incision inferius) to the NB line (mm)
22	Pg-NB	The perpendicular distance from pogonion to the NB line (mm)
23	SE	Distance between Sella and the foot point from the most posterior point of the condyle to the SN line (mm)
24	S-Ns-Sn	The angle between the S-Ns and Ns-Sn line (degrees)
25	S-Ns-Bs	The angle between the S-Ns and Ns-Bs line (degrees)
26	G-Sn-Pos	The angle between the G′-Sn and Sn-Pos line (degrees)
27	Ns-Prn-Pos	The angle between the Ns-Prn and Prn-Pos line (degrees)
28	NLA(Cm-Sn-UL)	The angle between the Cm-Sn and Sn-UL line (degrees)
29	AsUL-FH	The angle between the As-UL line and Frankfort horizontal plane (degrees)
30	BsLL-FH	The angle between the Bs-LL line and Frankfort horizontal plane (degrees)
31	AsUL-BsLL	The angle between the As-UL and Bs-LL line (degrees)
32	LL-Bs-Pos	The angle between the LL-Bs and Bs-Pos line (degrees)
33	Sn-Stoms	Distance between the subnasale and stomion superius (mm)
34	Stomi-Mes	Distance between the stomion inferius and soft tissue menton (mm)
35	Sn-Prn(FH)	The perpendicular distance from the pronasale to the line perpendicular to Frankfort horizontal plane through the subnasale (mm)
36	Ns-N(FH)	The perpendicular distance from nasion to the line perpendicular to the Frankfort horizontal plane through the soft tissue nasion (mm)
37	Sn-A (FH)	The perpendicular distance from subspinale to the line perpendicular to Frankfort horizontal plane through the subnasale (mm)
38	Bs-B(FH)	The perpendicular distance from the supramental to the line perpendicular to Frankfort horizontal plane through the most posterior point of mentolabial sulcus (mm)
39	ChinThickness	Distance between gnathion and the gnathion of soft tissue (mm)
40	UL-EP	The perpendicular distance from the upper labral to the E-line (pronaslae to pogonion of soft tissue) (mm)
41	LL-H	The perpendicular distance from the lower labral to the H-line (upper labral to pogonion of soft tissue) (mm)
42	LL-EP	The perpendicular distance from the lower labral to the E-line (pronasale to pogonion of soft tissue) (mm)

### 2.3 Statistical Analysis

**FIGURE 4:**
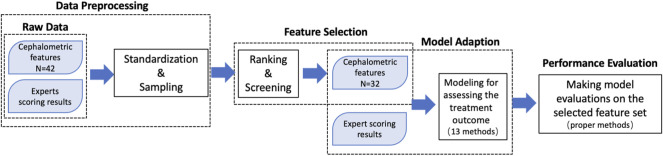
Diagram of the process of analyzing the quantitative evaluation, which was performed by utilizing the cephalometric features as input data and the expert evaluation scores as output data.

To evaluate the orthodontic treatment and fit it with the experts’ comprehensive scoring, the following steps were taken (Figure 4): 1) data preprocessing; 2) feature selection and model adaption; and 3) performance evaluation.

#### 2.3.1 Data Preprocessing

As mentioned previously, the cephalometric features and expert evaluation scores were utilized as input and output data. The first step was to make the data sets comparable. Before statistical analysis, data standardization was conducted through the z-score, as shown in Eq. 1. Here, $X=\left\{x_{i}\right\}$ was the cephalometric feature set for all subjects, and μ and σ represented the average value and standard deviation of the normal population in China (M and X, 1965; X et al., 1986).

$$Z\left(X\right)=\left|\left(x_{i}-\mu \right)/\sigma \right|,$$ (1)

**FIGURE 5:**
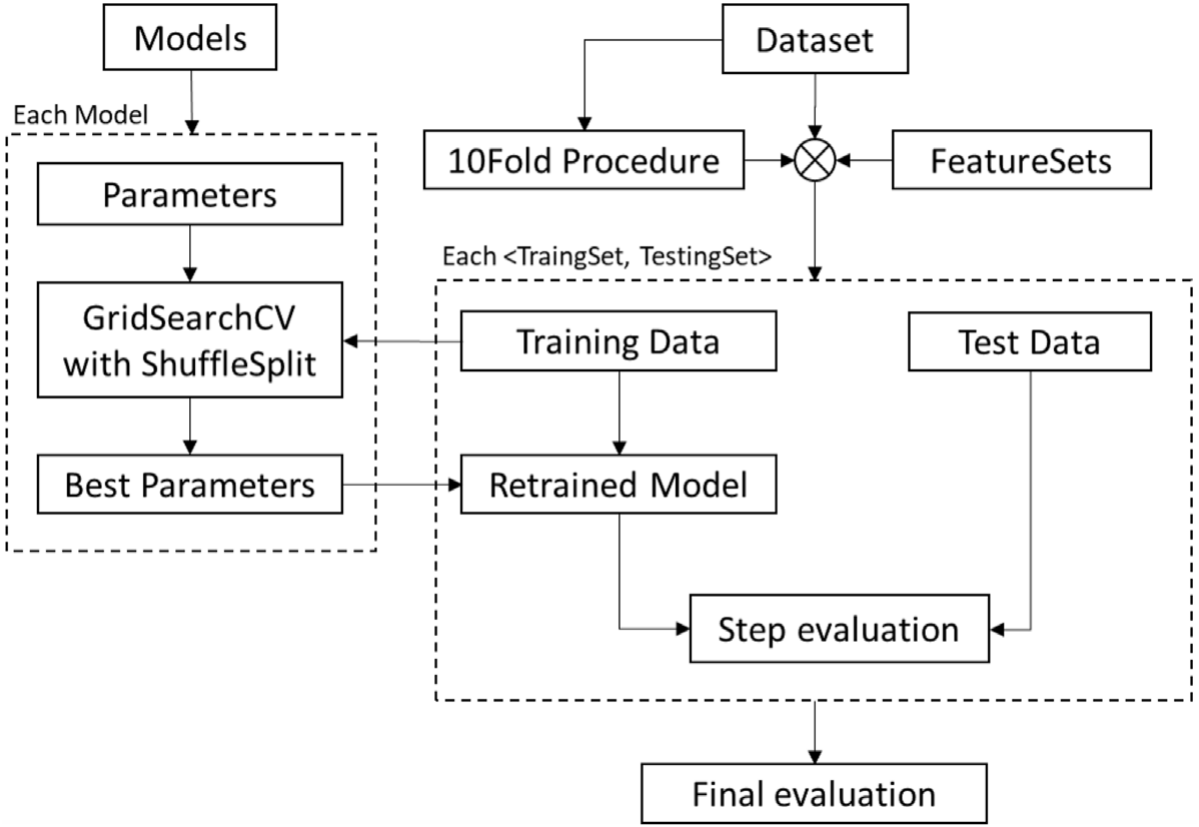
The flowchart of the cross-validation workflow from the scratch to evaluate the performance of each model after feature selection.

The data could be split into two subsets as follows: 1) a training set and 2) a testing set. When there are different settings (“hyperparameters”) in an estimator, the validation set is introduced to solve the “leaking”-overfitting issues on the testing set. However, it is not suitable for our scenarios of the small sample dataset, only containing 180 samples. Splitting into three subsets, the available training data will be further reduced for learning the model. To use the data efficiently, the procedure named cross validation (CV) is applied in our solution and the validation set is no longer needed. There are a lot of different ways to perform a CV. As shown in Figure 5, we have introduced two kinds of them in our solutions, which are the 10-fold procedure and the GridSearch CV with a ShuffleSplit function. We take the 10-fold procedure for evaluating per step in addition to each typical training–testing process because one evaluation on a small testing set of only 18 samples may not accurately reflect the performance of the entire model. The 10-fold procedure could allow for a fairer test as every sample has the same chance to be and to have been divided into the training set or the testing set, and the final evaluation takes the statistical indicators on all samples at the testing set. During the GridSearch CV process, the training samples from the 10-Fold procedure are first randomly shuffled, then split into a pair of training and testing sets, and lastly, sent to select hyperparameters, train the model, and evaluate the performance of the trained model. In the ShuffleSplit function, we set it five times for the shuffle division process and take 20% of the data as the testing set.

#### 2.3.2 Feature Selection and Model Adaption

Feature selection usually has two purposes and utilizes feature selection techniques. One technique is to reduce the clutter of original features, which includes highly correlated elements or irrelevant features. The other technique is to reduce the difficulty of analysis and increase prediction accuracy. This part of the work relies on feature engineering, which is based on the ranking of variable importance. The linear regression models often have the disadvantage of collinearity, which greatly affects error levels. In our solution, the initial feature screening was conducted to eliminate collinearity. The variable selection method was based on the correlation analysis (Ruz and Araya-Díaz, 2018) and was performed as follows:

The correlation analysis was performed on the 42 factors as well as the factors and the subjective outcomes (experts scoring results). One factor was retained out of two or more factors with Pearson correlation coefficients of 0.7 and above, with which the highest correlation with the subjective outcome was selected as the retention factor from these factors with collinearity.

**FIGURE 6:**
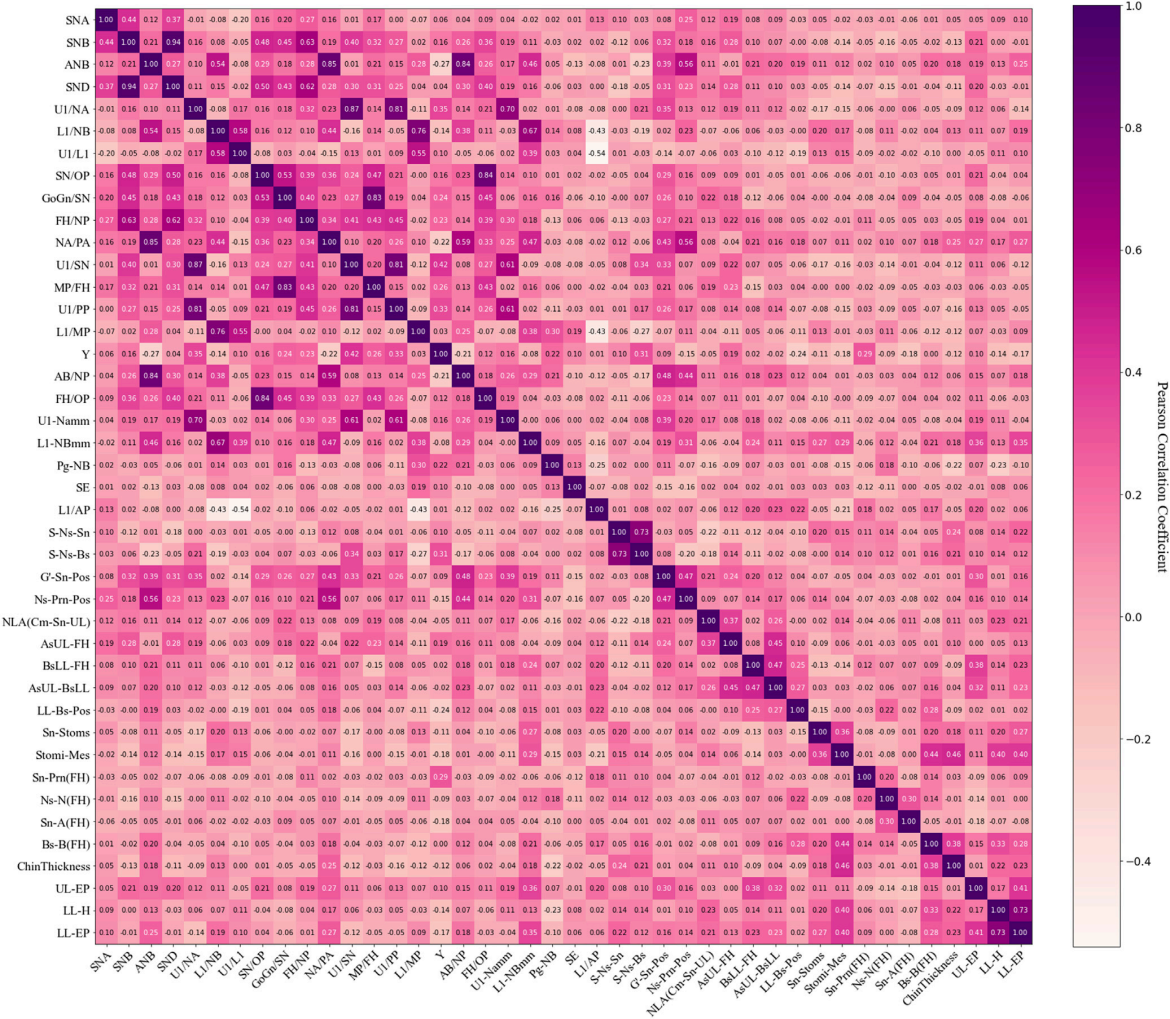
Pearson correlation coefficients with 42 factors.

Then, ten factors (SND, U1/NA, NA/PA, MP/FH, U1/PP, L1/MP, AB/NP, FH/OP, S-Ns-Sn, and LL-H) were removed, leaving 32 factors for the subsequent analysis (Figure 6).

For the model adaption procedure, the initial model screening was conducted to find relatively suitable algorithms from the common and widely used machine learning methods, which are listed in Table 2. The 13 algorithms in Table 2 comprise five categories of machine learning models including linear models, SVMs, decision trees, ANNs (artificial neural networks), and ensemble models, which are introduced in detail in Supplementary Material S2.

**TABLE 2:** The list of 13 methods from Scikit-learn.

Name	Description
sklearn.linear_model. LinearRegression^a^	It estimates the coefficients by applying Ordinary Least Squares
sklearn.linear_model.Lasso^a^	It can estimate sparse coefficients, which addresses the issue of the least-squares penalty minimization with the $\ell_{1}$ -norm of the coefficient vector
sklearn.linear_model.Ridge^a^	It introduces a penalty with $\ell_{2}$ -norm on the size of the coefficients to help solve the problem of collinearity
sklearn.tree. DecisionTreeRegressor^a^	It is a non-parametric supervised learning method to make predictions for a target variable by learning the decision rules inferred from the input features
sklearn. ensemble. GradientBoostingRegressor^a^	It supports a series of different loss functions. Here, we take the default loss function for regression, i.e., least squares
sklearn.ensemble. AdaBoostRegressor^a^	It assembles a sequence of weak learners with a weighted majority vote by taking the repeated boosting iteration
sklearn. ensemble. BaggingRegressor^a^	It introduces randomization into the construction procedure of an estimator and then makes an ensemble by splitting and aggregating individual predictions of this estimator on random subsets of the original training set
sklearn.ensemble. RandomForestRegressor^a^	It aims at decreasing the variance of the forest estimator by using bootstrap samples from the training set and random subsets of candidate features for node splitting
sklearn.ensemble. ExtraTreesRegressor^a^	It is similar to the random forests with node splitting. However, it randomly generates thresholds for each candidate feature and picks the best of these thresholds as the splitting rule
sklearn.neural_network. MLPRegressor^a^	It implements a multilayer perceptron (MLP) with no activation function in the output layer. Its output is a set of continuous values, and it takes the square error as the loss function
sklearn.svm.LinearSVR^a^	It is only suitable for the linear kernel when solving regression problems
sklearn.svm.SVR^a^	There are three kinds of kernels in this algorithm, i.e., linear, polynomial, and RBF kernels. Here, we take the RBF kernel
xgboost.XGBRegressor^b^	It implements the Scikit-Learn Wrapper interface for XGBoost regression

a https://scikit-learn.org/stable/supervised_learning.html.

b https://xgboost.readthedocs.io/en/latest/.

The mean absolute error (MAE) and root mean square error (RMSE) could be used to assess the fitting performance of the models, as shown in Eqs 2, 3. Here, $X=\left\{x_{i}\right\}$ is the cephalometric feature set of all subjects. n stands for the total number of X , which is 180 in our case. $\left\{y_{i}\right\}$ is the set of ground-truth scores corresponding to each subject. $f\left(x_{i}\right)$ is the analytical approach that takes the cephalometric features as inputs and the prediction values as outputs.

$$MAE\left(X,f\right)=\frac{1}{n}\overset{n}{\underset{i=1}{\sum }}\left|f\left(x_{i}\right)-y_{i}\right|,$$ (2)

$$RMSE\left(X,\;f\right)=\sqrt{\frac{1}{n}\sum_{i=1}^{n}{\left[f\left(x_{i}\right)-y_{i}\right]}^{2}},$$ (3)

#### 2.3.3 Performance Evaluation

**FIGURE 7:**
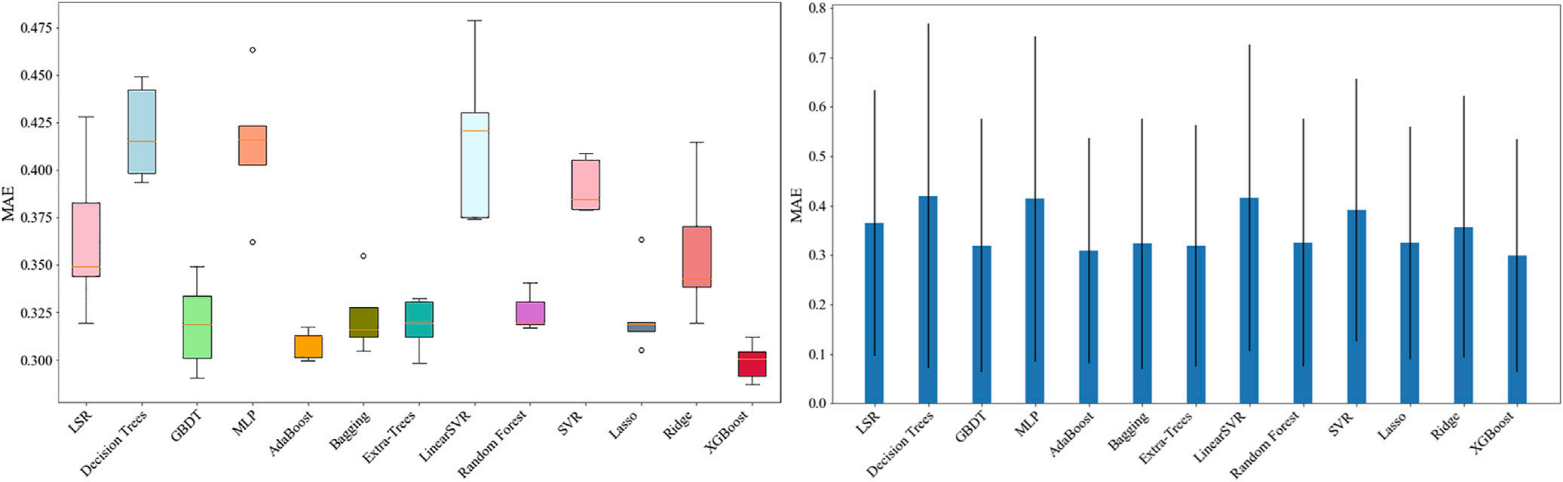
Results of the initial model screening: the MAE of AdaBoost, ExtraTrees, and XGBoost had the lowest values within the smallest standard deviations, compared with that of the other models.

Based on the performance in the data processing (seen in Figure 7), there are four subsequent approaches: AdaBoost, ExtraTrees, XGBoost, and LSR were selected to compare subsets of cephalometric features. Here, the reason for including LSR with a mediocre performance is that LSR is utilized as a conventional approach in most medical studies. Therefore, we utilized LSR as a baseline method. We choose the other three approaches for further comparisons because they have yielded stable and relatively small metric values.

Then, the MCCV method was applied again, splitting the samples into the training set and testing set 10 times at random. The 32 factors were sequentially incorporated into the models in terms of their ranking list according to the relevance to the experts’ evaluation scores. The mean absolute error (MAE), root mean square error (RMSE), and Pearson correlation coefficient were produced to assess the final fitting performance of the models.

## 3 Results

In this section, we evaluate the performance of four algorithms, i.e., AdaBoost, ExtraTrees, XGBoost, and LSR, by using the MCCV method. In this case, K-fold (K = 10) cross validation was applied. In the feature selection portion, we found that choosing a subset of no more than 32 features was the best option. Based on the importance rank order of these features, we gradually select feature subsets with a feature number from 1 to 32. Therefore, for each feature subset, we applied the MCCV method and took the average values of the model computing outcomes as the final results of the metrics. We will show the results from three aspects, including the model fitting performance, predicted performance, and model interpretability.

### 3.1 Model Fitting Performance

**FIGURE 8:**
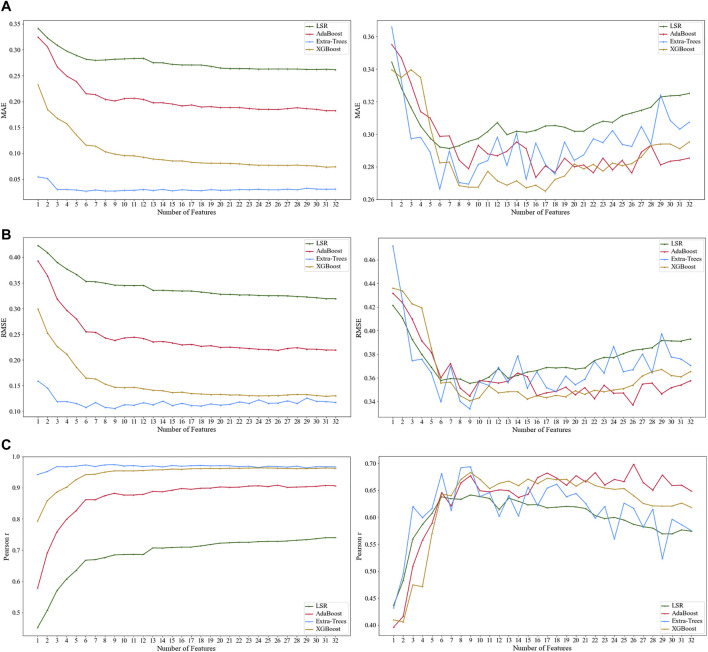
Results from the total sample by using XGBoost regression, ExtraTrees regression, AdaBoost regression, and linear regression: **(A1)** mean absolute error (MAE); **(B1)** root mean square error (RMSE); and **(C1)** Pearson correlation coefficient. The results from the testing set sample by using XGBoost regression, ExtraTrees regression, AdaBoost regression, and linear regression: **(A2)** mean absolute error (MAE); **(B2)** root mean square error (RMSE); and **(C2)** Pearson correlation coefficient.

The line chart (Figure 8) could show the trend in the model fitting performance when the features (from 1 to 32) were included in turn. Such a chart could help us screen for the suitable model. In particular, when the line chart jiggles dramatically, it often means that the method is severely over-fitted. When this phenomenon occurs, the method needs to be excluded, even though it may show ideal numerical results at some nodes.

Figures 8A1–C1 show the total sample. As the number of features entering the model increased, the values of MAE and RMSE of the XGBoost regression model (the yellow line) and ExtraTrees regression model (the blue line) were less than 0.2, and the Pearson correlation coefficient was also closer to 1, which indicated a better fitting performance compared to the other two methods.

Figures 8A2–C2 show the testing set, which can more sufficiently explain the performance problem. The overall trend of the line chart showed that the MAE and RMSE values decreased at the beginning as the number of features entering the model increased and then increased as the number of factors increased after reaching the trough. In particular, the XGBoost and ExtraTrees regression models (the yellow and blue lines) appeared more often at the troughs compared to the other two models.

However, the result of the ExtraTresss regression (the blue line) yielded a more pronounced curve jitter both in the total sample and testing set model, indicating a possible severe risk of overfitting. Therefore, the ExtraTrees regression model (the blue line) was not applicable to this study, and we ultimately concluded that the XGBoost model had better fitting performance.

### 3.2 Predicted Performance

To evaluate the predictive performance, the exact number of features should be clarified, when the best-predicted model is performed. Values of the MAE, RMSE, and Pearson correlation coefficient from the testing set were used for comparison among XGBoost regression, ExtraTress regression, AdaBoost regression, and linear regression. In Figure 8, as the method of XGBoost regression was selected in Section 3.1, three nodes (when 9, 15, and 17 features were included, respectively) were then picked for further comparison, as the values at the three nodes were near the peak or trough and were relatively close to each other.

Table 3 shows the results of the values of the performance indicators from those four methods. When nine features were included, the performance of the XGBoost regression model was MAE = 0.267, RMSE = 0.341, and Pearson correlation coefficient = 0.683; when 17 features were included, the performance of the XGBoost regression model was MAE = 0.265, RMSE = 0.343, and Pearson correlation coefficient = 0.672. Although the latter MAE value was slightly smaller than the former one, the former RMSE and Pearson correlation coefficient values were both better than those of the latter. Therefore, the XGBoost regression model exhibited the best predictive performance when nine features were included.

**TABLE 3:** Machine learning model performance in the testing set.

Number of features	Performance indicator	XGBoost regression		ExtraTrees regression		AdaBoost regression		Linear regression	
		Mean	SD	Mean	SD	Mean	SD	Mean	SD
9	MAE	0.267	0.077	0.269	0.061	0.279	0.071	0.296	0.071
	RMSE	0.341	0.086	0.334	0.074	0.345	0.078	0.355	0.075
	Correlation coefficient	0.683	0.163	0.694	0.133	0.677	0.153	0.641	0.161
15	MAE	0.267	0.077	0.272	0.077	0.291	0.071	0.301	0.061
	RMSE	0.342	0.094	0.351	0.084	0.361	0.078	0.365	0.071
	Correlation coefficient	0.671	0.186	0.656	0.155	0.642	0.148	0.623	0.165
17	MAE	0.265	0.071	0.281	0.081	0.281	0.069	0.305	0.062
	RMSE	0.343	0.092	0.352	0.099	0.347	0.079	0.369	0.073
	Correlation coefficient	0.672	0.168	0.654	0.200	0.682	0.160	0.617	0.164

### 3.3 Model Interpretability

**FIGURE 9:**
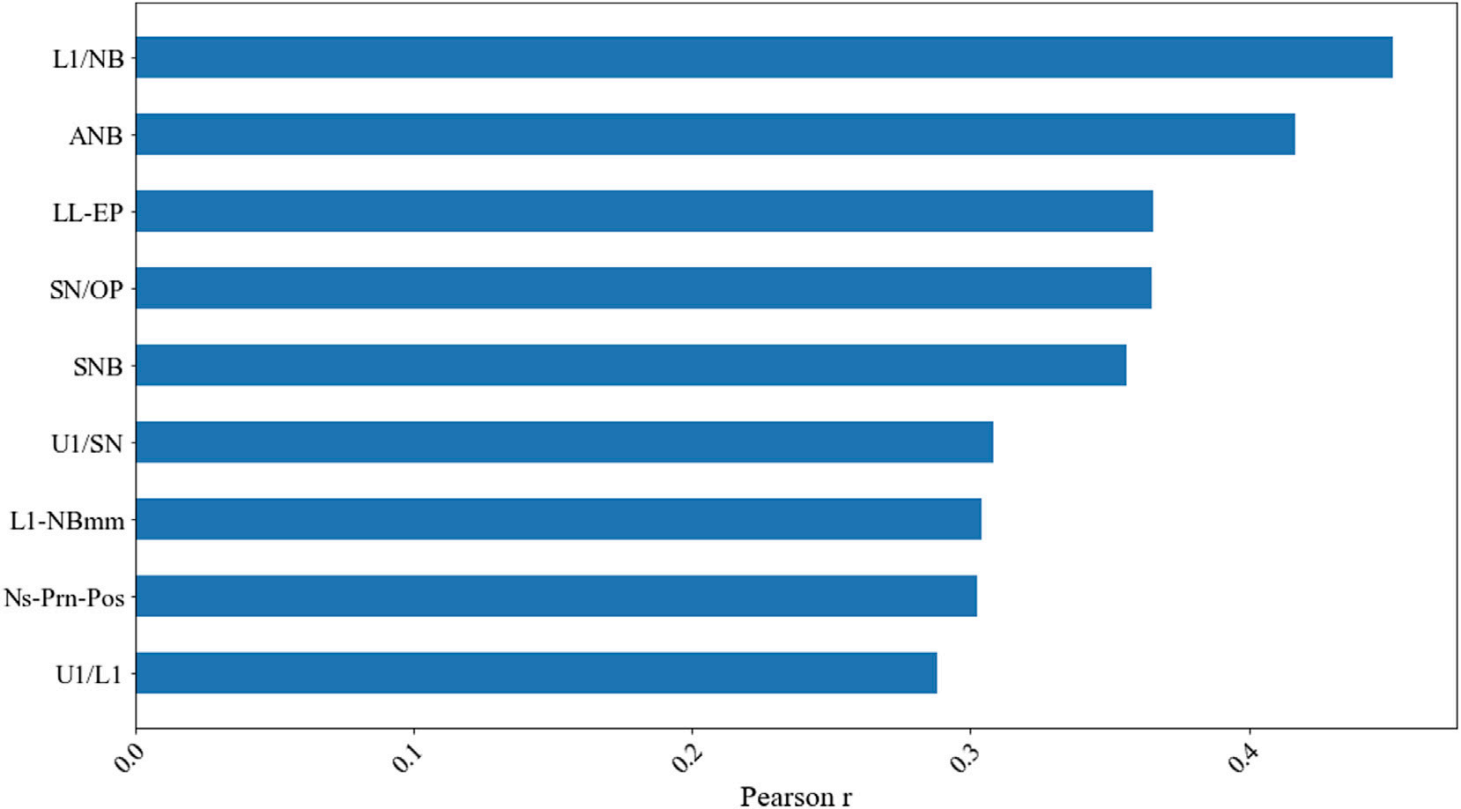
Results of the Pearson correlation coefficient from the testing set when nine factors were included by using the XGBoost regression model.

The results of the testing set were best predicted by the XGBoost regression method when nine features were entered into the model, including L1/NB, ANB, LL-EP, SN/OP, SNB, U1/SN, L1-NBmm, Ns-Prn-Pos, and U1/L1, which reflected the lower incisors, the anterior–posterior relationship between the upper and lower jaws, the prominence of the lower lip, the steepness of the occlusal plane, the anterior–posterior position of the lower jaw relative to the cranial base, the prominence of the lower incisors, the prominence of the nose, and the relative labial inclination of the upper and lower incisors (Figure 9).

## 4 Discussion

The recent advances in machine learning and a large amount of available data have laid the foundations to apply the machine learning methodology to various orthodontic aspects, including automated landmark detection on lateral cephalograms and photography images, facial attractiveness, and skeletal classification, as well as determining the degree of cervical vertebra maturation, providing orthodontic tooth extraction decisions, and predicting the need for orthodontic treatment or orthognathic surgery. Based on current studies, the most promising applications have been focused on predicting the need for treatment and decision making for tooth extractions before orthodontic treatment (Mohammad-Rahimi et al., 2021). However, the application to evaluate the craniodentofacial morphological harmony after orthodontic treatment attracts rare attention. Orthodontic treatment achieves the goal of improving the function, balance, and aesthetics of the hard and soft tissue structure by moving the teeth. Orthodontists have been working on methods to assess the results of orthodontic treatment and to be able to objectively assess the merits of treatment results, both on a case-by-case basis and a comparison between cases. The most widely used methods of outcome evaluation include the PAR (Peer Evaluation Rating) index, the ABO-OGS (American Board of Orthodontics-Objective Grading System) evaluation system, and the ICON (Index of Complexity, Outcome and Need), each of which has its own characteristics and should be used in the evaluation of orthodontic clinical cases within a certain field. However, these methods are based on research samples and practitioners from Europe and the United States, the developed statistical methods are limited, and no orthodontic outcome evaluation system has been established for Chinese patients. The present study proposed to apply a machine learning approach to evaluate the posttreatment cephalometric diagrams of patients for Chinese orthodontic specialists, presenting a methodological innovation and an analysis of the factors incorporated into the evaluation. The model also aimed to improve the prediction performance for facial profile congruence judgment after orthodontic treatment and to find the most important characteristics when evaluating cephalometric morphological harmony by orthodontists. The model learns from past routine measurements, either including or excluding the factors concluded from the 2D images of cephalometric diagrams and/or the 3D images of plaster casts of dentitions which are used to compute the orthodontic index. The proposed XGBoost regression model was shown to be effective and precise in handling this task by performing better than the other machine learning models and traditional statistical methods that predict the scores of experts. Compared with the other approaches reported in the literature (Yu et al., 2014, 2016), the major advantages of the proposed XGBoost regression model from the present study involve the ability to deal with lower and smaller transversal data sample size.

This study identified 9 out of 42 cephalometric factors (including L1/NB, ANB, LL-EP, SN/OP, SNB, U1/SN, L1-NB, Ns-Prn-Pos, and U1/L1) that significantly influenced the orthodontist judgments when evaluating posttreatment satisfaction and facial morphological harmony. The factors could then be categorized into the following parts: the tooth position, tooth alignment, jaw position, and soft tissue morphology. These four parts cannot be separated regarding the facial morphological harmony evaluation and are structurally interlinked and influenced by each other.

For the first part, the lower incisor inclination (L1/NB), lower incisor prominence (L1-NB), upper incisor inclination (U1/SN), and relative inclination of the upper and lower incisors (U1/L1) reflect the tooth position. Some researchers (Kambara et al., 2016) investigated how the position of mandibular incisors affected facial profile aesthetics and concluded that the position of mandibular incisors for Japanese patients should be within a Holdaway ratio of 2–3 (distance from L1 to NB divided by the distance from Pog to NB) when the distance from L1 to the NB line was considered. Uesato G et al. (Uesato et al., 1978) stated that 5 mm was an ideal figure for the distance from L1 to the NB line when Steiner analysis was applied to Japanese individuals. For the second part, the occlusal plane steepness (the angle between SN and the occlusal plane, SN/OP) reflected the vertical alignment of the teeth and, to some extent, the vertical facial type. The vertical alignment of the teeth corresponded to the occlusion of the teeth, which was determined to some extent by the vertical orientation of the facial type and the direction of the occlusal muscles. Anteroposterior and vertical facial type variations influenced the aesthetic preference of the anteroposterior lip positions and further influenced the judgement of facial harmony after orthodontic treatment. For the third part, the anteroposterior positions of the maxilla to the mandible (ANB) and the mandible to the cranial base (SNB) reflected the jaw position. For orthodontists, angle classification is the most widely used method of determining the sagittal occlusal relationship of the upper and lower teeth. Angle classification reflects to some extent skeletal malocclusion, which is the upper and lower jaw position relative to the skull. Patients with Angle Class I and Skeletal Class I (ANB = 2.7 ± 2°) usually have a normal jaw position, while patients with Angle Class II and Skeletal Class II often present with maxillary protrusion and mandibular retrusion, and patients with Angle Class III and Skeletal Class III present with maxillary retrusion and mandibular protrusion. However, with the different sagittal relationships of the upper and lower jaws, experts may develop different plans for orthodontic treatment regardless of the type of angle classification. Moreover, patients with different jaw positions may experience different difficulties and have different orthodontic treatment expectations (Turley, 2015). For the fourth part, nasal prominence/the total facial convexity angle (Ns-Prn-Pos) and lower lip prominence (lower lip to E-line, LL-EP) reflected the soft tissue morphology, which appeared to influence the aesthetics and harmony of the facial profile after orthodontic treatment. These are the features that represent the anteroposterior position of the lower lip and the amount of noise that influences the profile/facial convexity (Fortes et al., 2014). Some researchers (Perović, 2017) pointed out that the significant differences in profiles of people with class II division two compared to class I were the position of the lower and upper lip in relation to the S-line (which is another reference plane with a similar function as the E-line). Others (Joshi et al., 2015) found that the sagittal lip positions were associated with the skeletal malocclusion pattern.

The present study revealed the nine significant cephalometric features integrated into the abovementioned four parts which not only determine the most important characteristics when experts comprehensively evaluate various landmarks and components on cephalometric films but also provide evidence about the relationship among these characteristics. When assessing cases, experts may focus more on the correlation of these important factors, rather than just a standard value for a particular measurement.

Overall, the significance of our study was reflected in three main aspects: 1) first, it was the attempt to apply machine learning methods to the expert evaluation of craniodentofacial morphological harmony after orthodontic treatment, which was methodologically different from traditional statistical methods, and the results showed that the XGBoost regression model improved the fitting and predicted performance of the model over linear regression; 2) second, based on the model, we have taken the orthodontic clinical perspective to analyze the included features, which validated the content of the features of clinical interest from the machine learning perspective of our study; and 3) third, based on the first two aspects, this study provided ideas for future exploration of similar machine learning algorithms using small samples from orthodontic clinics.

Computers and technology continue to allow us to study, predict, and produce aesthetic results that were previously thought to be unattainable. Digitalized clinical databases stored in the form of photographs, lateral cephalometric films, CBCT, 3D models, and the associated software programs have improved our ability to analyze hard and soft tissue data. In future work, further studies need to focus on exploring new solutions or enhancing the ability to utilize automation.

## 5 Conclusion

Within the limitation of the present study, the practical application of the XGBoost regression model performed a better predictive ability than that of the other models regarding the cephalometric morphological harmony evaluation by experts after orthodontic treatment. The present methodology also provided guidance for the application of machine learning models to medical problems characterized by limited datasets sizes. Moreover, the teeth position, teeth alignment, jaw position, and soft tissue morphology were demonstrated to be the most significant factors that influenced the craniodentofacial morphological harmony judgment by orthodontists.

## Data Availability

The raw data supporting the conclusion of this article will be made available by the authors, without undue reservation.
