# Supplementary material for: Machine Learning-Based Evaluation on Craniodentofacial Morphological Harmony of Patients After Orthodontic Treatment
Source: Front Physiol. 2022 May 9;13:862847. doi: 10.3389/fphys.2022.862847 (PMC9124867; doi:10.3389/fphys.2022.862847)
Supplement: Supplementary file 1 [file DataSheet2.docx]

**Supplementary material 2**

**TABLE 1** The descriptive analysis of the scores from judges in the overlapping sample

| number | Panel of 69 judges | | Panel of 36 judges | |
| --- | --- | --- | --- | --- |
|  | mean | SD | mean | SD |
| A01 | 1.3188 | 0.6881 | 1.1111 | 0.3187 |
| A02 | 2.8116 | 0.6299 | 2.6388 | 0.4871 |
| A03 | 1.2464 | 0.4185 | 1.1666 | 0.4472 |
| A04 | 2.2174 | 0.5875 | 2.3333 | 0.6761 |
| A05 | 1.9130 | 0.6279 | 2.0277 | 0.6963 |
| A06 | 1.5797 | 0.7647 | 1.6666 | 0.6761 |
| A07 | 1.1594 | 0.6737 | 1.2500 | 0.5000 |
| A08 | 1.8841 | 0.6616 | 1.8055 | 0.7490 |
| A09 | 1.4928 | 0.4296 | 1.5277 | 0.6087 |
| A10 | 2.4493 | 0.4296 | 2.6666 | 0.4780 |
| A11 | 2.0145 | 0.5136 | 2.0000 | 0.5855 |
| A12 | 1.6087 | 0.3392 | 1.7222 | 0.5662 |
| E01 | 2.6377 | 0.7503 | 2.8888 | 0.3187 |
| E02 | 1.1884 | 0.6058 | 1.1666 | 0.4472 |
| E03 | 2.6377 | 0.6564 | 2.4444 | 0.6068 |
| E04 | 1.3623 | 0.6141 | 2.2222 | 0.6808 |
| E05 | 1.6812 | 0.5835 | 1.9166 | 0.8062 |
| E06 | 2.2899 | 0.4694 | 2.5277 | 0.6087 |
| E07 | 1.4493 | 0.6702 | 1.2500 | 0.5000 |
| E08 | 1.6087 | 0.5283 | 1.5555 | 0.5039 |
| E09 | 1.9710 | 0.2054 | 1.7777 | 0.6808 |
| E10 | 2.1739 | 0.7092 | 2.4444 | 0.6522 |
| E11 | 1.9710 | 0.7043 | 2.0000 | 0.5345 |
| E12 | 1.2609 | 0.7046 | 1.3333 | 0.5345 |

**TABLE 2** The descriptive analysis of the scores from judges in the two panels

| Sample | Panel of 69 judges | | Panel of 36 judges | |
| --- | --- | --- | --- | --- |
|  | mean | SD | mean | SD |
| Total | 1.8517 | 0.4661 | 1.8256 | 0.4917 |


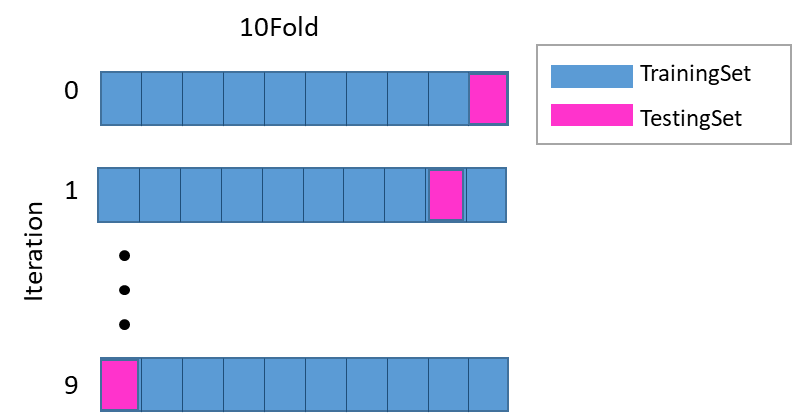

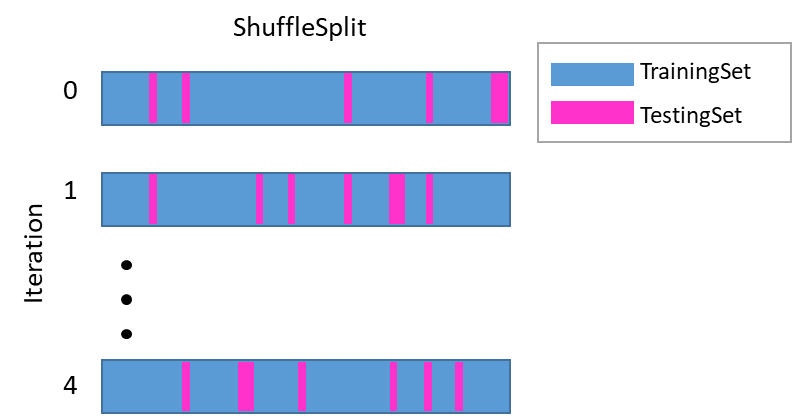


**FIGURE 1** Interation processing of 10-Fold (left) and ShuffleSplit (right)


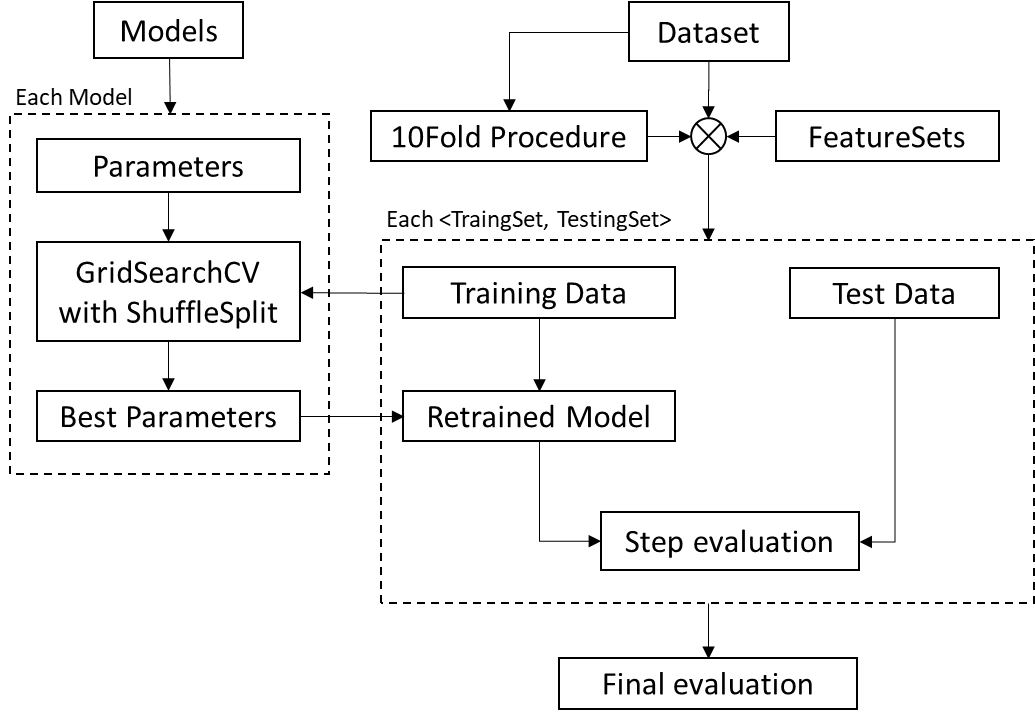


**FIGURE 2** The flowchart of our cross-validation workflow from scratch to evaluate the performance of each model after feature selection
