# Supplementary material for: Machine Learning-Based Evaluation on Craniodentofacial Morphological Harmony of Patients After Orthodontic Treatment
Source: Front Physiol. 2022 May 9;13:862847. doi: 10.3389/fphys.2022.862847 (PMC9124867; doi:10.3389/fphys.2022.862847)
Supplement: Supplementary file 2 [file DataSheet1.docx]

**Supplementary material 1**

In linear models, the target value is expected to be a linear combination of the input features. There are three linear models in the method set of our study, i.e., LSR, Ridge Regression, and Lasso. LSR fits a linear model by minimizing the residual sum of squares between real target values and predicted target values. Ridge regression solves several problems of least squares by including a penalty on the size of the coefficient, which makes it more robust to collinearity. Lasso (Kim et al., 2007) estimates sparse coefficients (fewer nonzero coefficients) by consisting of an added regularization term. Depending only on a subset of the training, SVMs (Vapnik, 1998) are still effective in cases where the number of features is greater than the number of samples. To solve regression problems, there is an extension of support vector classification, named support vector regression. SVR and LinearSVR in our method set are three different implementations of support vector regression, where the first two methods are similar but have slightly different sets of parameters, and the last one is a faster implementation for the case of a linear kernel. Decision trees (Hastie et al., 2009) are a nonparametric method, of which the goal is to learn simple decision rules inferred from features for predicting the value of the target variable in regression problems. In this work, an optimized version of the CART (classification and regression trees) is applied, which constructs binary trees using the feature and threshold. The basic unit of ANN (Ripley, 2008) is called node or neuron. There are three different types of nodes, i.e., input nodes, hidden nodes, and output nodes. MLP is the simplest version of ANN, which has only a hidden layer. To solve regression problems, there is no activation function in the output layer. Ensemble methods improve generalizability or (and) robustness by combining the predicted results of several base estimators. According to the fusion methods of base estimates, ensemble methods could be divided into two families, i.e., averaging methods and boosting methods. In the first family, base estimators are independently built and then average their outputs. In our work, there are three average methods in the method set, which include bagging, random forests, and extra-trees. A bagging regressor (Louppe and Geurts, 2012) fits the base regressor each on random subsets of the original dataset and then averages their individual predictions to obtain the final output. A random forest regressor (Breiman, 2001) improves the accuracy and control overfitting by averaging the outputs of a series of decision trees on subsamples of the dataset produced by bagging. An extra-trees regressor (Geurts et al., 2006) fits several randomized decision trees on various subsamples of the dataset and then obtains the average of them. In boosting methods, the driving principle is to build base estimators sequentially and try to reduce the bias of the whole model. There are also three methods in our method set, i.e., AdaBoost, GBDT and XGBoost. AdaBoost (Drucker, 1997) fits regressors on the original dataset iteratively, and in each iteration, the weights of instances are adjusted according to the result of its previous iteration. As such, difficult cases will receive increasing attention. Tree boosting is highly effective and widely used on various data science challenges. GBDT (Hastie et al., 2009) is a generalization of boosting constructing additive regression models by sequentially fitting a weak learner to current pseudoresiduals of the loss function at each iteration. Similar to other boosting algorithms, GBDT takes the exact greedy algorithm to find the best split on all the features. In GBDT, CART-based decision tree regressors of fixed size are used as weak learners. XGBoost (Chen and Guestrin, 2016), which implements the algorithms under the gradient boosting framework, is a sparsity-aware algorithm for approximate tree learning. It innovatively addresses the issue of finding quantiles on weighted data, which commonly exists in approximate tree boosting algorithms, by using the weighted quantile sketch. This approach has been widely applied to many machine learning problems and has achieved state-of-the-art performance.

**References**

Breiman, L. (2001). Random Forests. *Mach Learn* 45, 5–32. doi:10.1023/a:1010933404324.

Chen, T., and Guestrin, C. (2016). XGBoost: A Scalable Tree Boosting System. in *Proceedings of the 22nd ACM SIGKDD International Conference on Knowledge Discovery and Data Mining* KDD ’16. (New York, NY, USA: Association for Computing Machinery), 785–794. doi:10.1145/2939672.2939785.

Drucker, H. (1997). Improving Regressors Using Boosting Techniques. *Proceedings of the 14th International Conference on Machine Learning*.

Geurts, P., Ernst, D., and Wehenkel, L. (2006). Extremely randomized trees. *Mach Learn* 63, 3–42. doi:10.1007/s10994-006-6226-1.

Hastie, T., Tibshirani, R., and Friedman, J. (2009). *The Elements of Statistical Learning*. Springer doi:10.1007/978-0-387-84858-7.

Kim, S.-J., Koh, K., Lustig, M., Boyd, S., and Gorinevsky, D. (2007). An Interior-Point Method for Large-Scale ℓ1-Regularized Least Squares. *Ieee J Sel Top Signa* 1, 606–617. doi:10.1109/jstsp.2007.910971.

Louppe, G., and Geurts, P. (2012). Ensembles on Random Patches. in *Machine Learning and Knowledge Discovery in Databases*, eds. P. A. Flach, T. D. Bie, and N. Cristianini (Berlin, Heidelberg: Springer Berlin Heidelberg), 346–361.

Ripley, B. (2008). *Pattern Recognition And Neural Networks*. Cambridge University Press doi:10.1017/cbo9780511812651.

Vapnik, V. N. (1998). *Statistical Learning Theory*. New York: John Wiley & Sons.
